# Supplementary material for: Detailed molecular and epigenetic characterization of the pig IPEC-J2 and chicken SL-29 cell lines
Source: iScience. 2023 Feb 20;26(3):106252. doi: 10.1016/j.isci.2023.106252 (PMC10018572; doi:10.1016/j.isci.2023.106252)
Supplement: Data S2. Complete homer output for identified motifs in Chicken SL-29, related to Tables 5 and 6 — Homer motif analysis results for histone modifications H3K4me1, H3K4me3, H3K27ac, enhancers, and ATAC-seq of chicken SL-29 cell line. Parameters for possible false positives is as mentioned earlier for S5. [file mmc3.zip › Data_S2/S6/Chicken_SL_29/peak_files_H3K4me3/homerResults/motif3.similar.html]

motif3

## Information for motif3

T
A
C
G
A
C
T
G
A
C
G
T
C
T
A
G
G
T
C
A
A
C
T
G
A
G
C
T
T
C
A
G
  
Reverse Opposite:  

A
G
T
C
T
C
G
A
A
G
T
C
A
C
G
T
G
A
T
C
C
G
T
A
A
G
T
C
A
T
G
C
  

|  |  |
| --- | --- |
| p-value: | 1e-105 |
| log p-value: | -2.420e+02 |
| Information Content per bp: | 1.830 |
| Number of Target Sequences with motif | 4851.0 |
| Percentage of Target Sequences with motif | 28.26% |
| Number of Background Sequences with motif | 6914.6 |
| Percentage of Background Sequences with motif | 21.20% |
| Average Position of motif in Targets | 149.6 +/- 83.3bp |
| Average Position of motif in Background | 150.3 +/- 92.3bp |
| Strand Bias (log2 ratio + to - strand density) | 0.1 |
| Multiplicity (# of sites on avg that occur together) | 1.20 |
| Motif File: | file (matrix) reverse opposite |

### Similar de novo motifs found

|  |  |  |  |  |  |  |  |
| --- | --- | --- | --- | --- | --- | --- | --- |
| Rank | Match Score | Redundant Motif | P-value | log P-value | % of Targets | % of Background | Motif file |
| 1 | 0.853 | T G C A T C A G A C T G A C G T C T G A C G T A A T C G A T G C T C A G T A C G | 1e-91 | -211.504460 | 14.36% | 9.49% | motif file (matrix) |
| 2 | 0.630 | T C G A T A C G A T C G A T G C T G A C A G T C C A G T G T A C T G C A T A C G | 1e-79 | -183.108174 | 24.45% | 18.62% | motif file (matrix) |
| 3 | 0.791 | T G C A G A T C A C T G T G A C G C T A A T G C C G A T A G T C G T C A A G T C | 1e-67 | -156.534325 | 46.70% | 40.11% | motif file (matrix) |
| 4 | 0.630 | A T C G A T C G G C A T C A T G G C T A C T A G C A T G C A T G G T A C G A T C C T A G A T G C T A C G | 1e-59 | -137.632888 | 30.98% | 25.42% | motif file (matrix) |
| 5 | 0.734 | A T C G A C T G T A G C A T G C G A T C A T C G A G T C C G T A A G T C A G C T A G T C C G T A A G T C | 1e-49 | -113.423454 | 8.53% | 5.72% | motif file (matrix) |
| 6 | 0.720 | A C T G T A C G C A G T A C T G T G C A A C T G A T G C C T A G T A C G A T G C T A G C A G C T T A C G | 1e-31 | -71.863230 | 3.10% | 1.79% | motif file (matrix) |
| 7 | 0.701 | T A C G C A G T T A C G C T G A T A C G C A G T T A C G C T G A T C A G T A G C T C A G T C G A T A C G | 1e-19 | -44.502254 | 2.03% | 1.20% | motif file (matrix) |
| 8 | 0.669 | G A T C G T C A T A G C T C G A A T G C C T G A G A T C T C G A | 1e-14 | -32.351313 | 20.24% | 17.96% | motif file (matrix) |
